# Association of Appendicitis Incidence With Warmer Weather Independent of Season

**DOI:** 10.1001/jamanetworkopen.2022.34269

**Published:** 2022-10-03

**Authors:** Jacob E. Simmering, Linnea A. Polgreen, David A. Talan, Joseph E. Cavanaugh, Philip M. Polgreen

**Affiliations:** 1 Department of Internal Medicine, University of Iowa, Iowa City; 2 Department of Pharmacy Practice and Science, University of Iowa, Iowa City; 3 Ronald Reagan UCLA (University of California, Los Angeles) Medical Center, Department of Emergency Medicine, UCLA; 4 Department of Biostatistics, University of Iowa, Iowa City; 5 Department of Epidemiology, University of Iowa, Iowa City

## Abstract

**Importance:**

Acute appendicitis is a common cause of abdominal pain and the most common reason for emergency surgery in several countries. Increased cases during summer months have been reported.

**Objective:**

To investigate the incidence of acute appendicitis by considering local temperature patterns in geographic regions with different climate over several years.

**Design, Setting, and Participants:**

This cohort study used insurance claims data from the MarketScan Commercial Claims and Encounters Database and the Medicare Supplemental and Coordination of Benefits Database from January 1, 2001, to December 31, 2017. The cohort included individuals at risk for appendicitis who were enrolled in US insurance plans that contribute data to the MarketScan databases. Cases of appendicitis in the inpatient, outpatient, and emergency department settings were identified using *International Classification of Diseases, Ninth Revision, Clinical Modification* or *International Statistical Classification of Diseases, Tenth Revision, Clinical Modification* diagnosis codes. Local weather data were obtained for individuals living in a metropolitan statistical area (MSA) from the Integrated Surface Database. Associations were characterized using a fixed-effects generalized linear model based on a negative binomial distribution. The model was adjusted for age, sex, and day of week and included fixed effects for year and MSA. The generalized linear model was fit with a piecewise linear model by searching each 0.56 °C in temperature for change points. To further isolate the role of temperature, observed temperature was replaced with the expected temperature and the deviation of the observed temperature from the expected temperature for a given city on a given day of year. Data were analyzed from October 1, 2021, to July 31, 2022.

**Main Outcomes and Measures:**

The primary outcome was the daily number of appendicitis cases in a given city stratified by age and sex, with mean temperature in the MSA over the previous 7 days as the independent variable.

**Results:**

A total of 450 723 744 person-years at risk and 689 917 patients with appendicitis (mean [SD] age, 35 [18] years; 347 473 male [50.4%] individuals) were included. Every 5.56 °C increase in temperature was associated with a 1.3% increase in the incidence of appendicitis (incidence rate ratio [IRR], 1.01; 95% CI, 1.01-1.02) when temperatures were 10.56 °C or lower and a 2.9% increase in incidence (IRR, 1.03; 95% CI, 1.03-1.03) for temperatures higher than 10.56 °C. In terms of temperature deviations, a higher-than-expected temperature increase greater than 5.56 °C was associated with a 3.3% (95% CI, 1.0%-5.7%) increase in the incidence of appendicitis compared with days with near-0 deviations.

**Conclusions and Relevance:**

Results of this cohort study observed seasonality in the incidence of appendicitis and found an association between increased incidence and warmer weather. These results could help elucidate the mechanism of appendicitis.

## Key Points

**Question:**

Is appendicitis incidence associated with ambient temperature?

**Findings:**

In this cohort study of 689 917 patients with appendicitis, every 5.56 °C increase in temperature was associated with a 1.3% increase in the incidence of appendicitis at temperatures 10.56 °C or lower and a 2.9% increase in incidence at temperatures higher than 10.56 °C.

**Meaning:**

Findings of this study suggest that the incidence of appendicitis increases when the temperature increases, independent of season.

## Teaser

This cohort study examines the association of temperature, seasonal factors, and behavioral changes with higher incidence of appendicitis across the US.

## Introduction

Acute appendicitis is a common cause of abdominal pain^1^ and is the most common reason for emergency surgery in several countries.^2^ An estimated 250 000 appendectomies are performed each year in the US alone,^3^ and the overall lifetime risk for acute appendicitis is between 7% and 8%.^2^ In past decades, new diagnostic and treatment approaches have been developed, including prediction scores,^4,5,6^ ultrasonographic imaging and reduced-dose computed tomography,^7,8^ and laparoscopic surgery,^9,10^ along with nonoperative treatments for uncomplicated cases that use antibiotic therapy alone.^10,11^ In contrast to these diagnostic and treatment innovations, little progress has been made toward understanding the risk factors for this syndrome, and its precise etiology remains unclear.

A better understanding of the risk factors for appendicitis may provide insights into the pathogenesis of appendicitis. However, to date, few risk factors have been described.^12,13,14,15,16,17,18,19,20,21,22,23,24,25,26,27^ Acute appendicitis most commonly occurs between age 10 and 30 years,^12,13^ with a slight preponderance in male individuals.^14,15^ There appears to be a genetic predisposition for developing appendicitis among individuals with a family history of this condition.^16^ Dietary risk factors may include low fiber diet,^17,18^ increased sugar intake,^19,20^ and decreased water consumption.^21,22^ Environmental risk factors may include exposure to air pollution,^23^ allergens,^24^ cigarette smoke,^25^ and gastrointestinal infections.^26^ However, one of the most commonly observed environmental risk factors is the seasonal pattern in several countries, wherein the incidence of acute appendicitis increases during the summer months.^12,27^

A potential explanation for the higher incidence in summer is that warmer weather could increase the risk for dehydration and subsequent constipation.^28^ Other behaviors potentially associated with the increased risk for acute appendicitis in summer months may include changes in diet and exposure to gastrointestinal pathogens.^26^ Furthermore, previous studies of the seasonality of acute appendicitis did not specifically examine temperature, coarse temperature measurements (eg, monthly averages^28,29^), or large and climate-diverse geographic regions.^29,30,31,32,33^ Not all past studies found an increase in the incidence of acute appendicitis in the summer.^33^ The objective of the present study was to investigate the incidence of acute appendicitis by considering local temperature patterns in geographic regions with different climate over several years.

## Methods

This cohort study was deemed as nonhuman participant research by the University of Iowa Institutional Review Board, which waived the informed consent requirement. We followed the Strengthening the Reporting of Observational Studies in Epidemiology (STROBE) reporting guideline.

### Data Source

We used data from the MarketScan Commercial Claims and Encounters Database and the Medicare Supplemental and Coordination of Benefits Database (IBM Corp) from January 1, 2001, to December 31, 2017. These databases contain insurance claims data for individuals enrolled in participating insurance plans across the US. We identified cases of appendicitis using *International Classification of Diseases, Ninth Revision, Clinical Modification* or *International Statistical Classification of Diseases, Tenth Revision, Clinical Modification* diagnosis codes (eTable 1 in the Supplement) in any setting, including outpatient, inpatient, and emergency department. If a person had multiple dates with an appendicitis diagnosis, we retained only the first date.

On a given day, we considered all individuals in the MarketScan databases as being at risk for appendicitis. Approximately 80% of these individuals lived in 1 of approximately 400 metropolitan statistical areas (MSAs). We excluded individuals who did not live in an MSA or in the contiguous US because we could not obtain local weather data for these people.

Weather data were obtained from the Integrated Surface Database published by the National Centers for Environmental Information, part of the National Oceanic and Atmospheric Administration. The Integrated Surface Database contains hourly weather observations at more than 35 000 weather stations throughout the world from 1901 to the present. We used weather data from 1990 onward. We defined the weather for an MSA by using temperature readings from all of the weather stations within 100 km (approximately 62 miles) of the centroid of the MSA and then calculating a simple mean temperature.

### Statistical Analysis

The primary outcome was the daily number of cases of appendicitis in a given city stratified by age and sex, with the primary independent variable being mean temperature in the MSA over the previous 7 days. We elected to use mean temperature over mean high or low temperature during the previous 7 days because both high daytime and high nighttime temperatures matter; that is, the daytime heat is as important as whether heat relief occurs during the overnight hours. We selected 7 days because we expected a short period between exposure (higher temperatures) and appendicitis presentation. A risk window of 7 days seemed certain to capture the true risk window, with allowance for delayed presentation owing to holidays or weekends but without oversmoothing by being too long. We controlled for age, sex, and day of week in the model and included fixed effects for year and MSA. Race and ethnicity data were not available in the MarketScan databases.

Although cities are different from each other (eg, residents in Miami, Florida, may spend more time outdoors during the winter than residents in Fargo, North Dakota), they generally do not change year to year. The MSA fixed effect allows for differences between cities but assumes that a particular city’s difference remains constant over time. We included a year fixed effect to reduce confounding from year-to-year changes in medical practice (eg, point-of-care adoption of ultrasonography) or in insurance plans that contribute data to the MarketScan databases and thus affect all cities in the study. We divided age into ordinal groups (0-5, 6-10, 11-15, 16-20, 21-30, 31-40, 41-50, 51-60, 61-70, 71-80, and ≥81 years) and evaluated temperature as a linear spline using the R package lspline (R Foundation for Statistical Computing). We used a fixed-effects generalized linear model with a negative binomial distribution and a log-link function. The negative binomial family is useful for modeling counts, such as the number of cases per day, in which we expect that the mean does not equal the variance of the counts (overdispersion).

We included an offset of the log number of people who were enrolled on that given date of that given age and sex in that given MSA to account for the varying populations across MSAs and time. We also reported robust SEs with clustering by MSA.^34^

We established the number and placement of the change point in an iterative manner. First, we evaluated the fixed-effects model with no change point, obtaining an entirely linear change in log incidence with temperature. We used the Hannan-Quinn information criterion (HQC) as the measure of model performance.^35^ The HQC is similar to other information criteria, such as the Akaike information criterion or the bayesian information criterion, but uses a different penalty term for model complexity. Briefly, we were concerned that the Akaike information criterion, which assigns a fixed penalty of 2 for each parameter added to the model, would overfit the large sample and that the bayesian information criterion, which adds log(n) as a penalty, would underfit the sample. The HQC uses a penalty of 2 log(log(n)) and may provide a better balance of goodness of fit and model parsimony. Smaller HQC values indicated better model performance. The model with no changes in slope had an HQC of 5 649 821.

Second, we fit a series of models with 1 knot that varied by 0.56 degree, from –9.44 °C to 29.44 °C (the 1st to 99th percentile of observed temperature), and found the model with the best performance. We found that a change in the slope at 10.56 °C was associated with decreased HQC of 23.9 to 5 649 797.

Third, we considered adding a second change in slope. We estimated a series of models with a knot at 10.56 °C and with each 0.56 degree from –9.44 °C to 29.44 °C. We found that a change in the slope at 10.56 °C and 28.89 °C was associated with the lowest HQC of 5 649 798, but this value was larger than the model with only 1 slope change. We accepted as the final linear spline a model with a single change in slope at 10.56 °C.

A major limitation of the model was that it was not adjusted for seasonality. It is possible that omitted seasonal factors, as opposed to temperatures, were associated with the results. Simple approaches, such as including a series of month indicators in the model, were not appropriate because of the high collinearity between temperature and month. Instead, we replaced observed temperature with expected temperature for that given city on that given day of year and the deviation of observed temperature from expected temperature.

We determined the expected temperature by fitting a collection of regression models. Specifically, for each MSA, we regressed daily temperature on day-of-week and sine or cosine seasonality terms. With the sine or cosine expression, we assumed that temperature varied across the year smoothly and ensured that the end of December resembled the start of January. We used 1990 and later data in the Integrated Surface Database to build this temperature-estimation model. The difference between the observed temperature and the expected temperature from this model was the temperature deviation. Next, we computed the 7-day moving mean of temperature deviation to cover the risk window.

We estimated a model that replaced the temperature variable with 2 measures: temperature expected in that given city over the previous week and a series of binned deviations from expected temperature (eg, >5.56 °C lower, 2.78-5.56 °C lower, 1.67-2.78 °C lower, 1.11-1.67 °C lower, 0.56-1.11 °C lower, 0-0.56 °C lower, 0-0.56 °C higher, 0.56-1.11 °C higher, 1.11-1.67 °C higher, 1.67-2.78 °C higher, 2.78-5.56 °C higher, or >5.56 °C higher). We treated the expected temperature as a piecewise linear model using knots at the same location used in the primary model.

In addition, we assessed whether different appendicitis severity levels (with any peritonitis, without peritonitis, or other appendicitis) were associated with temperature. For all 3 levels of severity, we fit a model to characterize the association between the response and observed temperature.

All models were created using R, version 4.0.4 (R Foundation for Statistical Computing), and fixed-effects estimation was performed with the R package fixest. The threshold of statistical significance was 0.05. Data were analyzed from October 1, 2021, to July 31, 2022.

## Results

There were 547 231 910 person-years at risk for appendicitis in the MarketScan databases, of which 451 174 481 (82.4%) were in an MSA. After restricting to MSAs in the contiguous US with weather data, we included 450 723 744 person-years at risk and a total of 689 917 cases of appendicitis (mean [SD] age, 35 [18] years; 342 444 female [49.6%] and 347 473 male [50.4%] individuals). A summary of the age and sex distribution of the at-risk cohort and cases is provided in eTable 2 in the Supplement. We had 50 326 316 strata (unique combinations of age, sex, city, and date) across 736 different MSA values and 17 years. There were approximately 400 MSAs in the data set at any given time; however, the coding of the MSAs changed during data collection, giving us 736 unique values. Each MSA was followed for a mean (SD) period of 8.5 (4.4) years and contributed a median (IQR) of 191 863 (49 697-512 521) person-years of data.

**Figure 1.**
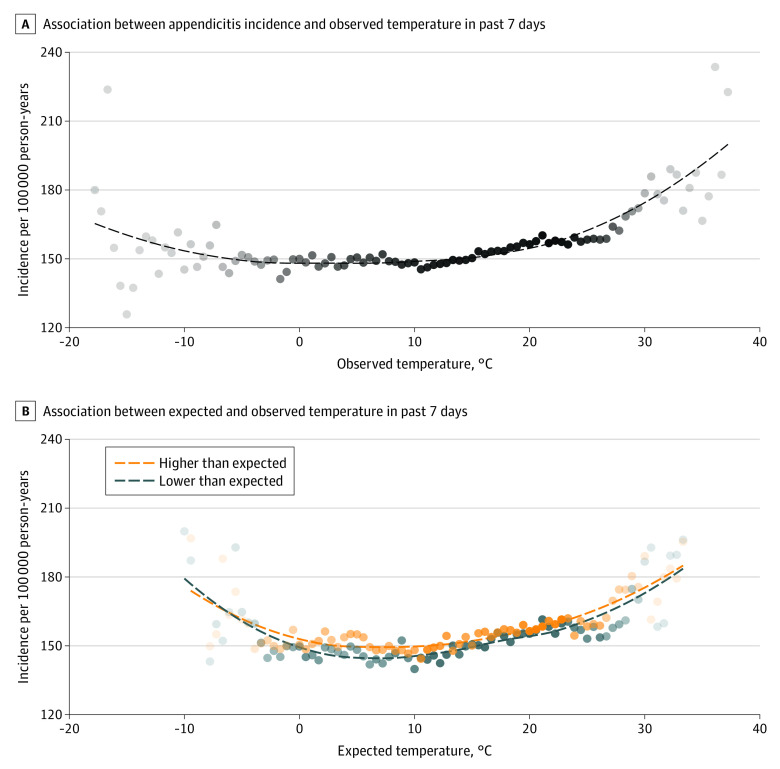
Incidence of Appendicitis by Previous Week Temperature Points are shaded according to the number of person-years at risk in that stratum. Dashed line represents a smooth estimate of the mean incidence at each temperature.

**Figure 2.**
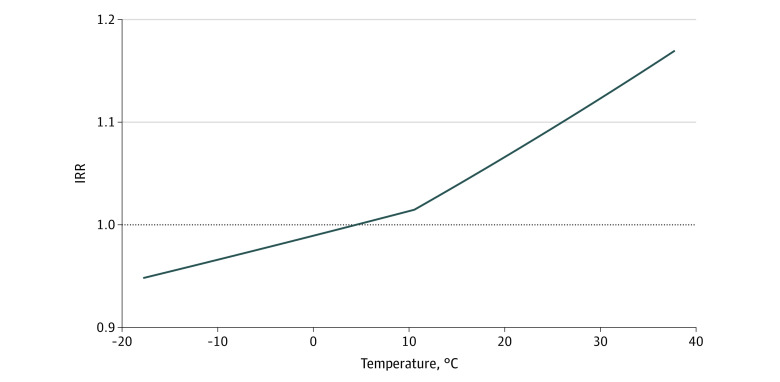
Estimated Outcome of Temperature in Piecewise Linear Model The solid line represents the estimated fit of the linear spline model for temperatures ranging from –17.78 °C to 37.78 °C. Note the change of slope at 10.56 °C. Reference was 4.44 °C. IRR indicates incidence rate ratio.

Compared with the temperature over the previous week, there was a dose-dependent increase in incidence of appendicitis, especially on days with temperatures higher than −12.22 °C (Figure 1). The linear spline in the fixed-effects model included a slope change at 10.56 °C, a value that seemed plausible in this unadjusted graph (Figure 2).

After adjustment for age, sex, day of week, year, and MSA, we observed an association between temperature and the incidence of appendicitis (Table 1). For every 5.56 °C increase in temperature, there was a 1.3% increase in incidence when temperatures were 10.56 °C or lower (incidence rate ratio [IRR], 1.01; 95% CI, 1.01-1.02) and a 2.9% increase for temperatures higher than 10.56 °C (IRR, 1.03; 95% CI, 1.03-1.03).

**Table 1.** Estimated Incidence Rate Ratios (IRRs) for Primary Model of Association of Temperature With Appendicitis Incidence

Factor	IRR (95% CI)
Sex	
Male	1 [Reference]
Female	0.92 (0.91-0.93)
Age, y	
0-5	[Reference]
6-10	4.63 (4.47-4.80)
11-15	7.43 (7.08-7.79)
16-20	8.53 (8.05-8.03)
21-30	7.44 (7.02-7.89)
31-40	6.32 (5.97-6.69)
41-50	5.09 (4.82-5.38)
51-60	4.40 (4.15-4.65)
61-70	3.87 (3.67-4.09)
71-80	3.37 (3.20-3.56)
≥81	2.78 (2.63-2.93)
Day of week	
Sunday	1 [Reference]
Monday	1.48 (1.46-1.50)
Tuesday	1.47 (1.46-1.49)
Wednesday	1.42 (1.40-1.44)
Thursday	1.38 (1.37-1.40)
Friday	1.35 (1.33-1.36)
Saturday	0.99 (0.98-1.00)
Previous week temperature, per 5.56 °C	
Slope at ≤10.56 °C	1.01 (1.01-1.02)
Slope at >10.56 °C	1.03 (1.03-1.03)

**Figure 3.**
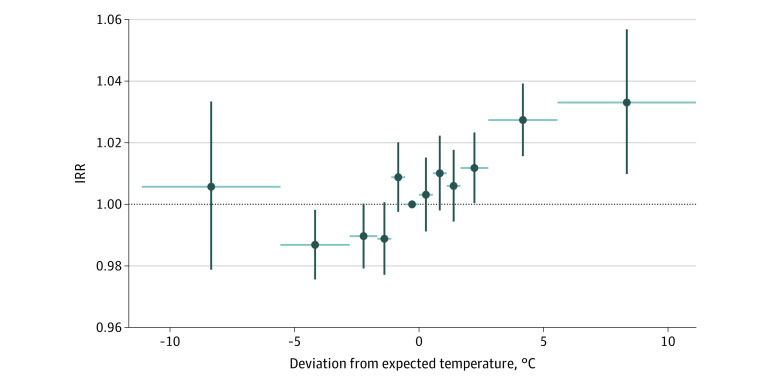
Estimated Association of Temperature Deviation With Incidence of Appendicitis Model was adjusted for year and metropolitan statistical area fixed effects, age, sex, day of week, and expected temperature. Vertical lines denote the 95% CIs using robust SEs adjusted for metropolitan statistical area clusters, and horizontal lines denote the size of the temperature bin. A dose-response association was observed between deviations in observed temperature from expected temperatures and incidence of appendicitis, with days after higher-than-normal-temperature periods having increased risk and days after lower-than-normal-temperature periods having decreased risk. Reference was –0.56 to 0 °C higher-than-expected temperature. IRR indicates incidence rate ratio.

The expected temperature model provided a high-quality fit to the observed temperatures across the US (eFigure 1 in the Supplement). The distribution of temperature deviations was similar between cities, following a normal distribution with an SD of approximately 2.78 °C (eFigure 2 in the Supplement). Before adjustment, warmer periods were consistently associated with higher incidence than cooler periods with the same expected temperature (Figure 1). After adjustment, we found a day after a week with more than 5.56 °C higher-than-expected temperature (approximately 2 SDs) that had a 3.3% (95% CI, 1.0%-5.7%) increase in incidence compared with a similar day after a week with 0 °C to 0.56 °C lower-than-expected temperature. The estimated incidence increased in a dose-dependent manner with higher deviation from expected temperature (Figure 3). Similarly, the cooler the day was compared with normal, the lower the incidence of appendicitis. A full description of the estimated coefficients is included in eTables 3 and 4 in the Supplement.

The observed association changed little when the model was stratified by disease severity (Table 2). In all 3 series (with any peritonitis, without peritonitis, or other appendicitis), warmer weather was associated with a similar increase in incidence. For every 5.56 °C increase in temperature, there was a 2.2% increase in incidence (IRR, 1.02; 95% CI, 1.01-1.02) for patients with peritonitis and a 2.5% increase in incidence (IRR, 1.02; 95% CI, 1.02-1.02) for patients without peritonitis when temperatures were 10.56 °C or lower. There was a 3.2% increase (IRR, 1.03; 95% CI, 1.03-1.04) for patients with peritonitis and a 4.4% increase (IRR, 1.04; 95% CI, 1.03-1.04) for patients without peritonitis for temperatures higher than 10.56 °C.

**Table 2.** Estimated Incidence Rate Ratios by Disease Severity

Previous week temperature, per 5.56 °C	Incidence rate ratio (95% CI)^**a**^		
	Appendicitis with peritonitis	Appendicitis without peritonitis	Other appendicitis
≤10.56 °C	1.02 (1.01-1.02)	1.02 (1.02-1.02)	1.01 (1.00-1.01)
>10.56 °C	1.03 (1.03-1.04)	1.04 (1.03-1.04)	1.03 (1.02-1.03)

^a^ Based on robust SEs adjusting for metropolitan statistical area clusters. All models included metropolitan statistical area and year fixed effects and were adjusted for age, sex, and day of week. See eTables 3 and 4 in the Supplement for full coefficient estimates.

## Discussion

Results of this cohort study found that the incidence of acute appendicitis not only was seasonal but also was associated with higher temperatures. We observed an association between appendicitis incidence and temperature over the previous week. Every 5.56 °C increase in temperature was associated with a higher incidence of appendicitis, which increased with the temperature during the previous week. This association was observable both in the unadjusted analysis and after adjustment for MSA, year, and demographic characteristics. The linear spline describing the role of temperature had a knot at 10.56 °C, a biologically plausible inflection point. Mean temperature of 10.56 °C featured daily highs between 15.56 °C and 21.11 °C, the lower bound of what most people consider to be warm weather. Specifications using mean high or mean low temperatures yielded similar results to those of a model using mean temperature.

In addition, we found that the incidence of appendicitis was associated with temperature that was higher than normal for a particular day. Specifically, when we assessed the deviations from expected temperature, we observed a dose-response pattern similar to that in the initial analysis. Examining the association between the risk of appendicitis and deviations from expected temperature could reduce potential confounding by omitting seasonal factors: deviations were small, more or less random changes in exposure. A typical July day in St Louis, Missouri, is approximately 26.67 °C, with highs of approximately 32.22 °C and lows of approximately 21.11 °C, and the temperature in July from year to year is relatively similar. However, a few days with high temperatures near 37.78 °C or mid-26.67 °C would be expected in any given month. These deviations—the slightly cooler or slightly warmer days—serve as a test of association that is not confounded by seasonality. When comparing incidence to deviation from expected temperature, we found a dose-response association: 5.56 °C warming was associated with a 3.3% increase in incidence compared with days with near-0 deviation. The presence of a dose-response pattern with a plausible exogenous measure of exposure suggests that temperature is associated with appendicitis incidence.

Most previous research has shown an increase in acute appendicitis incidence during summer months,^26^ but it has been difficult to separate warmer weather from other seasonal exposures (eg, gastrointestinal infections) or potential seasonal behavioral changes (eg, changes in diet). By examining a large geographic area (ie, entire continental US) for a period of 17 years, however, we were able to demonstrate not only the seasonality and an association between appendicitis incidence and weather but also the association of deviations from expected temperature with increased risk for acute appendicitis. Specifically, we were able to isolate the implications of temperature from the implications of seasonal factors and behavioral changes. Furthermore, this finding was robust for model selection. Temperature deviations remained consistent and substantial when using different model specifications with increasingly flexible allowances for seasonality.

Previous investigators have hypothesized that the seasonality of acute appendicitis is associated with dehydration and constipation attributed to warmer weather.^17,26^ In addition, people with a low-fiber diet are at 30% greater risk of appendicitis.^17^ Thus, warmer weather combined with a low-fiber diet may be associated with even higher levels of risk. Exposures to higher temperatures may also interact with genetic host factors for developing appendicitis. However, results of this study suggested that warmer weather was not associated with increased risk for specific age groups or for men or women. Instead, the risk associated with warmer weather appeared to apply to both sexes across the life span and to appendicitis cases with or without peritonitis.

### Limitations

This study has several limitations. First, the analysis was based on administrative data; diagnostic codes have limitations in terms of sensitivity and specificity. Second, we were unable to identify levels of personal exposure to the outside air temperature or access to air conditioning. Third, by using a fixed-effects model, we protected against time-invariant confounding among cities; however, cities and their populations may change over the study period. Detailed, individual-level exposure history is required to address some of these limitations. Fourth, we were not able to locate the approximately 20% of enrollees who did not reside in an MSA, and this cohort may lack rural representation as a result. Fifth, risk for acute appendicitis is most certainly multifactorial; thus, future investigation of the association of weather and/or dehydration with the incidence of appendicitis needs to capture potential genetic and dietary risk factors.

## Conclusions

This cohort study found an association between the increased incidence of appendicitis and warmer weather. In addition, the implications of weather in this association were isolated from those of other seasonal and behavioral factors, and risk of appendicitis during periods with higher-than-expected temperature was found to be higher. These results may help elucidate the mechanism of appendicitis.

## Notes

Supplement. **eTable 1.** ICD-9-CM and ICD-10-CM Codes Used to Identify Cases of Appendicitis **eTable 2.** Description of Cases and Cohort at Risk by Age and Sex **eFigure 1.** Model Fit for Each Metropolitan Statistical Area (MSA) **eFigure 2.** Temperature Deviations for Each Metropolitan Statistical Area (MSA) **eTable 3.** Full Model Estimates for Deviations Model—Binned Deviations **eTable 4.** Full Model Estimates Stratified by Severity—Observed Temperature
