# Supplementary material for: Association of Appendicitis Incidence With Warmer Weather Independent of Season
Source: JAMA Netw Open. 2022 Oct 3;5(10):e2234269. doi: 10.1001/jamanetworkopen.2022.34269 (PMC9530968; doi:10.1001/jamanetworkopen.2022.34269)
Supplement: Supplement. — eTable 1. ICD-9-CM and ICD-10-CM Codes Used to Identify Cases of Appendicitis eTable 2. Description of Cases and Cohort at Risk by Age and Sex eFigure 1. Model Fit for Each Metropolitan Statistical Area (MSA) eFigure 2. Temperature Deviations for Each Metropolitan Statistical Area (MSA) eTable 3. Full Model Estimates for Deviations Model—Binned Deviations eTable 4. Full Model Estimates Stratified by Severity—Observed Temperature [file jamanetwopen-e2234269-s001.pdf]

Supplemental Online Content

Simmering JE, Polgreen LA, Talan DA, Cavanaugh JE, Polgreen PM. Association of appendicitis incidence with warmer weather independent of season. *JAMA Netw Open*. 2022;5(10):e2234269. doi:10.1001/jamanetworkopen.2022.34269

eTable 1. ICD-9-CM and ICD-10-CM Codes Used to Identify Cases of Appendicitis

eTable 2. Description of Cases and Cohort at Risk by Age and Sex

eFigure 1. Model fit for each metropolitan statistical area (MSA)

eFigure 2. Temperature deviations for each metropolitan statistical area (MSA)

eTable 3. Full Model Estimates for Deviations Model—Binned Deviations

eTable 4. Full Model Estimates Stratified by Severity—Observed Temperature

This supplemental material has been provided by the authors to give readers additional information about their work.

© 2022 Simmering JE et al. *JAMA Network Open.*

eTable 1. ICD-9-CM and ICD-10-CM Codes Used to Identify Cases of Appendicitis

ICD Version	Diagnosis Code	Long Description
9	540.0	Acute appendicitis with generalized peritonitis
9	540.1	Acute appendicitis with peritoneal abscess
9	540.9	Acute appendicitis without mention of peritonitis
9	541	Appendicitis, unqualified
9	542	Other appendicitis
10	K35.2	Acute appendicitis with generalized peritonitis
10	K35.3	Acute appendicitis with localized peritonitis
10	K35.80	Unspecified acute appendicitis
10	K35.89	Other acute appendicitis
10	K37	Unspecified appendicitis
10	K36	Other appendicitis

© 2022 Simmering JE et al. *JAMA Network Open.*

eTable 2. Description of Cases and Cohort at Risk by Age and Sex

	Female Enrollees			Male Enrollees		
Age	Cases	Person-Years at Risk in 100,000s	Annualized Incidence Per 100,000	Cases	Person-Years at Risk in 100,000s	Annualized Incidence Per 100,000
0-5	3,365	130.5	25.8	4,306	136.9	31.4
6-10	15,110	137.3	110.1	22,156	143.3	154.6
11-15	27,137	152.1	178.4	38,968	158.5	245.8
16-20	36,918	159.8	231.0	42,472	165.2	257.0
21-30	59,764	293.1	203.9	60,093	270.1	222.5
31-40	58,002	333.0	174.2	55,515	297.9	186.4
41-50	56,699	390.1	145.4	50,374	349.0	144.3
51-60	51,523	397.2	129.7	42,110	350.2	120.3
61-70	22,424	205.3	10.92	20,583	184.6	111.5
71-80	7,349	84.9	86.6	7,376	69.9	105.5
81+	4,153	60.5	68.7	3,520	37.7	93.3

© 2022 Simmering JE et al. *JAMA Network Open.*

eFigure 1. Model fit for each metropolitan statistical area (MSA)

Panel A shows the R^2 of temperature prediction model for each of the MSAs in the IBM Marketscan data universe. Each point is one MSA in the IBM Marketscan data universe. Model fits were highest quality in the Northeast and Midwest states where seasonal patterns far exceed normal temperature variance and smallest in coastal cities where the normal variance is temperature is large relative to the seasonal pattern. Panel B shows a histogram of the model estimated R^2 values.

A

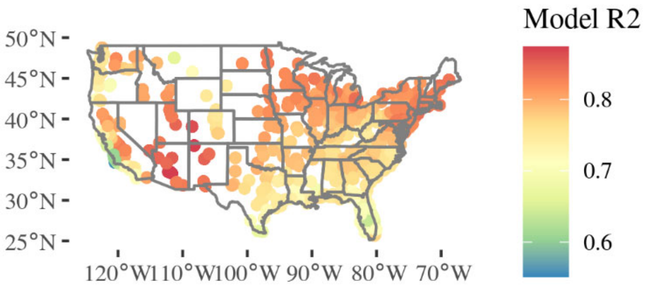

B

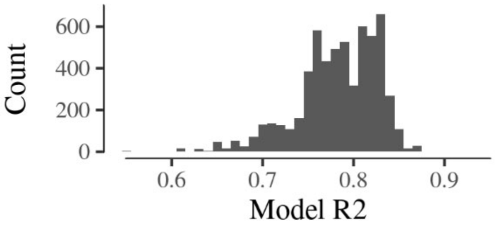

© 2022 Simmering JE et al. *JAMA Network Open.*

eFigure 2. Temperature deviations for each metropolitan statistical area (MSA)

The distribution of deviations was relatively similar across the different MSAs. Each line is the estimated density for the temperature deviations for one MSA. The majority of the MSAs clearly cluster together with a standard deviation of approximately 2.78°C.

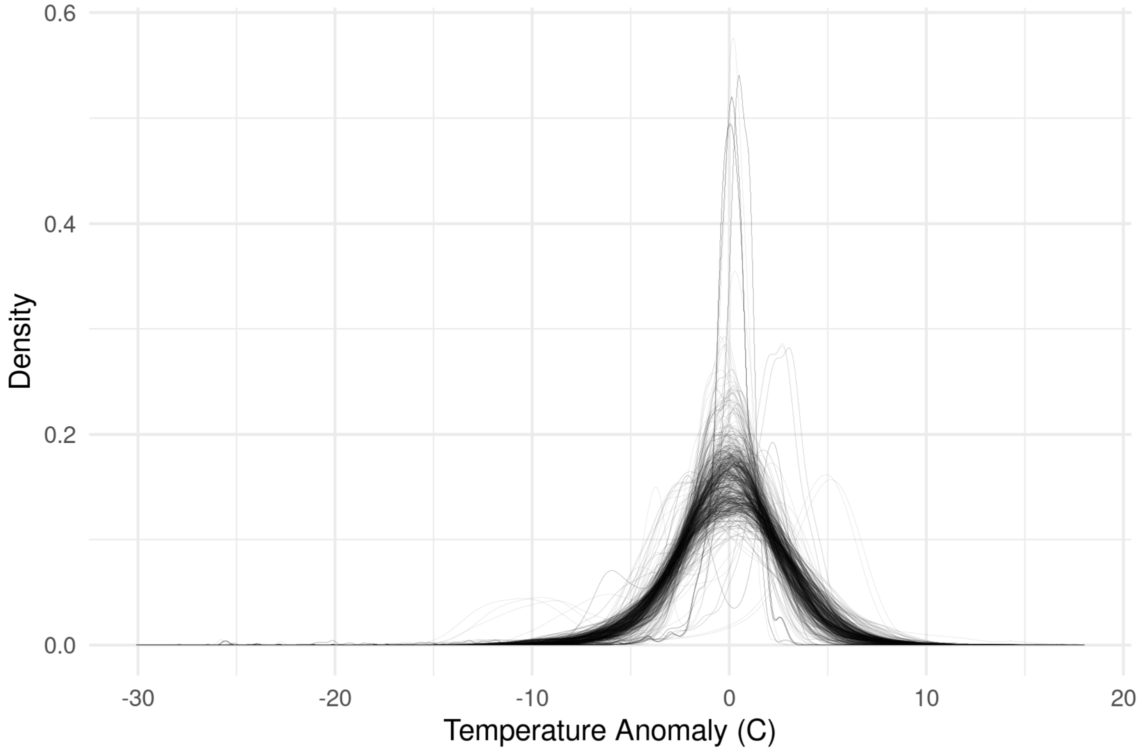

© 2022 Simmering JE et al. *JAMA Network Open.*

eTable 3. Full Model Estimates for Deviations Model—Binned Deviations

		95% CI (robust SE by clustered by MSA)	
	Incidence Rate Ratio	Lower Bound	Upper Bound
Female Sex	0.918	0.911	0.925
Age			
0-5	Reference		
6-10	4.630	4.469	4.796
11-15	7.426	7.082	7.786
16-20	8.525	8.049	9.029
21-30	7.441	7.019	7.888
31-40	6.323	5.974	6.691
41-50	5.091	4.820	5.377
51-60	4.394	4.153	4.650
61-70	3.870	3.665	4.086
71-80	3.372	3.194	3.560
81+	2.776	2.632	2.929
Day of Week			
Sunday	Reference		
Monday	1.477	1.458	1.497
Tuesday	1.473	1.456	1.491
Wednesday	1.422	1.404	1.440
Thursday	1.384	1.366	1.403
Friday	1.347	1.331	1.364
Saturday	0.987	0.975	0.999
Expected Prior Week Temperature			
Less than 10.56C	1.013	1.009	1.018
More than 10.56C	1.030	1.027	1.034
Deviation from Expected Temperature in °C			
Cooler than -5.56	1.006	0.979	1.033
-5.56 to -2.78	0.987	0.976	0.998
-2.78 to -1.67	0.990	0.979	1.000
-1.67 to -1.11	0.989	0.977	1.001
-1.11 to -0.56	1.009	0.998	1.020
-0.56 to 0	Reference		
0 to +0.56	1.003	0.991	1.015
0.56 to 1.11	1.010	0.998	1.022
1.11 to 1.67	1.006	0.994	1.018
1.67 to 2.78	1.012	1.000	1.023
2.78 to 5.56	1.027	1.016	1.039
Warmer than 5.6	1.033	1.010	1.057

© 2022 Simmering JE et al. *JAMA Network Open.*

eTable 4. Full Model Estimates Stratified by Severity—Observed Temperature

	With Peritonitis	Without Peritonitis	Other Appendicitis
Female Sex	0.793 (0.785, 0.802)	0.869 (0.862, 0.875)	0.926 (0.917, 0.935)
Age			
0-5	Reference	Reference	Reference
6-10	5.348 (5.116, 5.591)	9.738 (9.22, 10.286)	7.413 (7.022, 7.825)
11-15	4.834 (4.579, 5.103)	11.51 (10.787, 12.282)	8.074 (7.565, 8.618)
16-20	4.088 (3.864, 4.325)	10.061 (9.423, 10.743)	7.149 (6.677, 7.654)
21-30	3.921 (3.708, 4.147)	8.416 (7.901, 8.965)	6.098 (5.702, 6.521)
31-40	3.905 (3.698, 4.123)	6.589 (6.195, 7.009)	4.893 (4.586, 5.221)
41-50	3.636 (3.487, 3.791)	5.811 (5.567, 6.066)	4.77 (4.558, 4.991)
51-60	4.181 (3.957, 4.417)	5.506 (5.17, 5.865)	4.211 (3.933, 4.509)
61-70	4.28 (4.043, 4.532)	4.663 (4.377, 4.967)	3.618 (3.382, 3.871)
71-80	4.132 (3.902, 4.375)	3.699 (3.477, 3.934)	3.064 (2.872, 3.269)
81+	3.727 (3.523, 3.942)	2.823 (2.662, 2.993)	2.364 (2.208, 2.532)
Day of Week			
Sunday	Reference	Reference	Reference
Monday	1.391 (1.360, 1.423)	1.390 (1.373, 1.407)	1.559 (1.529, 1.590)
Tuesday	1.374 (1.346, 1.402)	1.381 (1.366, 1.397)	1.556 (1.528, 1.585)
Wednesday	1.307 (1.283, 1.332)	1.335 (1.319, 1.351)	1.501 (1.475, 1.527)
Thursday	1.289 (1.262, 1.318)	1.301 (1.285, 1.317)	1.462 (1.436, 1.490)
Friday	1.253 (1.228, 1.279)	1.265 (1.250, 1.280)	1.414 (1.389, 1.440)
Saturday	0.989 (0.969, 1.008)	0.980 (0.968, 0.992)	0.992 (0.977, 1.008)
Prior Week Temperature (per 5.56°C)			
Below 10.56°C	1.016 (1.008, 1.024)	1.019 (1.015, 1.023)	1.008 (1.002, 1.013)
Above 10.56°C	1.032 (1.026, 1.038)	1.036 (1.032, 1.041)	1.028 (1.022, 1.034)

© 2022 Simmering JE et al. *JAMA Network Open.*
